# Associations Between Online Learning, Smartphone Addiction Problems, and Psychological Symptoms in Chinese College Students After the COVID-19 Pandemic

**DOI:** 10.3389/fpubh.2022.881074

**Published:** 2022-05-04

**Authors:** Chi Zhang, Jinjuan Hao, Ye Liu, Ju Cui, Hao Yu

**Affiliations:** ^1^The Key Laboratory of Geriatrics, Beijing Institute of Geriatrics, Institute of Geriatric Medicine, Chinese Academy of Medical Sciences, Beijing Hospital, National Center of Gerontology of National Health Commission, Beijing, China; ^2^Hospital Administration Office, Beijing Hospital, National Center of Gerontology, Institute of Geriatrics Medicine, Chinese Academy of Medical Sciences, Beijing, China; ^3^Department of Education, Beijing Hospital, National Center of Gerontology, Institute of Geriatrics Medicine, Chinese Academy of Medical Sciences, Beijing, China

**Keywords:** online learning, problematic smartphone use, mental health, COVID-19, college students

## Abstract

**Background:**

Smartphone-based online education gained popularity during and after the COVID-19 pandemic. Although recent studies have highlighted the association between problematic smartphone use (PSU) and mental health symptoms, the potential role of online learning in this relationship remains unclear. This study aimed **t**o analyze the relationships between higher education modes, PSU, and related psychological symptoms in university students.

**Methods:**

A total of 1,629 Chinese university students from five provinces completed a web-based questionnaire survey between March 2020 and October 2021. Demographic characteristics and learning conditions were recorded. All participants completed the Smartphone Addiction Scale-Short Version, Patient Health Questionnaire, Generalized Anxiety Disorder Scale, and Athens Insomnia Scale. Multiple regressions models and stratified analyses were used to examine the association between online education mode, PSU, and symptoms of depression, anxiety, and insomnia.

**Results:**

The prevalence of PSU was 58.5%. Students who relied primarily on online learning had a higher prevalence of depressive symptoms (29.95% vs. 22.24%), anxiety symptoms (25.13% vs. 18.91%), and insomnia symptoms (75.89% vs. 70.27%) than those who relied on traditional face-to-face learning (*Ps* < 0.05). After adjusting for covariates, subjects with PSU were more likely to report depressive symptoms (AdjOR = 3.14, 95% CI = 2.26–4.37), anxiety symptoms (AdjOR = 3.73, 95% CI = 2.13–4.59), and insomnia symptoms (AdjOR = 2.96, 95% CI = 2.23–3.92) than those without PSU. Furthermore, the associations of PSU with depressive symptoms (OR = 4.66 vs. 2.33, *P* for interaction = 0.015) and anxiety symptoms (OR = 6.05 vs. 2.94, *P* for interaction = 0.021) were more pronounced in the online learning group.

**Conclusion:**

Our study provides preliminary evidence that Chinese university students have serious smartphone addiction problems, which are associated with depressive, anxiety, and insomnia symptoms. Online learning is found to exacerbate PSU and mental health problems. Our findings provide valuable information for targeted psychological interventions in the post-COVID-19 era.

## Introduction

The COVID-19 pandemic, a global public health emergency, has had significant impacts on China's healthcare and higher education system (1). The emergence of SARS-CoV-2 Delta and Omicron variants, currently spreading worldwide, has given rise to a new wave of the pandemic (2). On March 11th, 2020, the World Health Organization declared that the COVID-19 outbreak was a global pandemic, and governments have since implemented a series of strict social isolation policies to control the novel coronavirus (3). To avoid the spread of coronavirus among students, universities have adopted a variety of teaching methods, among which online learning has rapidly gained popularity (4). Online education has been found to significantly reduce the COVID-19 infection rate in students, and to effectively maintain the normal higher education teaching activities (5). Recent evidence suggested that the advancements of digital technology supported the effectiveness of online education (6).

However, just like every coin has two sides, online learning has some disadvantages. Other than the quality of education organization, scholars have also focused on the hidden negative effects of online leaning, such as Internet addiction and related mental health problems (7, 8). Both cross-sectional and longitudinal studies have confirmed that different types of Internet-related addiction behaviors were associated with psychological distress among mainland Chinese students during the COVID-19 school suspension (9, 10). Survey data have revealed that education supervision and related psychological problems are challenges that have yet to be overcome in the future education progress (11). While online learning is more flexible than traditional face-to-face learning, it increases the frequency of smartphone use among students. Recent studies have reported increased smartphone addiction and mental health problems since the COVID-19 outbreak (12, 13), yet it remains unclear whether online education contributes to this.

Since the COVID-19 outbreak in Wuhan in December 2019, the Chinese government has implemented effective social distancing measures, and students were forced to keep away from universities. Since the summer of 2020, most Chinese university students have been released from quarantine and returned to school. During and after the pandemic, a growing number of colleges have been implementing a transition from traditional face-to-face teaching methods to online teaching or a hybrid teaching, which has become the new normal. Researchers have expressed mixed views about the online education mode. Pei and Wuin concluded that online learning could enhance knowledge and skills to a greater degree than face-to-face learning (14). However, Hasan and Bao reported that the sudden transformation to distance learning was a challenge for students, and significantly exacerbated students' psychological stress (15). The severity of the COVID-19 pandemic has varied in different countries and areas. Despite the fact that China has effectively brought the coronavirus pandemic under control, new sporadic and imported COVID cases continue to emerge across the country. Thus, higher education methods must be tailored to these indeterminate epidemic conditions. For example, if there are no new cases in an area for a long time, local universities can maintain face-to-face and centralized teaching; however, if a new coronavirus epidemic occurs, universities must replace face-to-face teaching with online teaching until the local epidemic is effectively controlled.

During the COVID-19 pandemic, smartphones have played a vital role for health care and higher education progress (16), and university students have relied more on the Internet and smartphones for both social and learning activities (16). Some psychologists have regarded smartphone addiction and related psychological problems as a new hidden crisis of the COVID-19 pandemic (17). Recent systematic reviews have described the psychological distress and related influence factors during COVID-19 (18, 19). Over the past 2 years, several studies also reported the associations between COVID-19 psychological problems and smartphone addiction (20–22). In a large cross-sectional survey that included 89,588 Chinese college students, 41.1% reported anxiety symptoms (23). Recent studies have also revealed that the intensity and time of electronic device usage increased during the COVID-19 pandemic, and was positively correlated with various mental health problems (24, 25). Another national survey conducted by Ma et al. with 746,217 Chinese college students found that the prevalence of probable acute stress, depressive symptoms, and anxiety symptoms were 34.9, 21.1, and 11%, and the risks of developing depression and anxiety disorders among the student sample increased with the exposure time to electronic devices (26). Sleep problems appear to have been common during the ongoing COVID-19 pandemic. In Alimoradi et al.'s recent meta-analysis, sleep problems were found to be associated with higher levels of psychological distress (27). In one of our published studies, problematic smartphone use (PSU) was found to be associated with fatigue symptoms and sleep quality in medical students (28). However, limited research has examined the different mental health patterns caused by online education, especially in China. The present study will fill this gap in the literature by examining the potential role of online learning in the relationship between PSU and related mental health outcomes.

Many mechanistic studies have explored the relationship between PSU and clinical health outcomes, and specific theories have highlighted the potential effects of learning environment factors on health outcomes in student samples. PSU affects mental health through a variety of complex pathways, leading to symptoms of depression, anxiety, and loneliness (29). Some well-established theoretical frameworks are benefit for explaining the association of psychopathological symptoms with PSU. One of the most recognized theoretical frameworks is the Interaction of Person-Affect-Cognition-Execution (I-PACE) model (30). According to the I-PACE model, individuals' demographic background and psychological status, such as personality, emotional, cognitive, and executive functions can significantly predict Internet or smartphone addiction. As described by the Compensatory Internet Use Theory (CIUT), if individuals are in a negative situation, they may escape reality by surfing the internet, thus increasing the chance of addiction symptoms (31). The CIUT theory also places an emphasis on the importance of environmental factors in addiction risk. For instance, when college students rely primarily on the Internet for learning, they enter a more isolated social environment. Excessive smartphone use can lead to increased exposure to blue and short electromagnetic waves, which results in eye symptoms, sleep disturbances, and physical fatigue (25). In addition, online learning students are often less likely to receive effective educational supervision and timely assistance (32). Thus, the physical and mental health problems of these students should be paid more attention. Based on the existing theoretical mechanisms and relevant research findings, it seems possible that education mode is associated with PSU and mental health in university students. We thus proposed the following research hypotheses:

*Hypothesis 1 (H1)*. PSU is positively associated with levels of depression, anxiety, and insomnia symptoms.

*Hypothesis 2 (H2)*. Different learning modes are associated with different PSU levels in university students.

*Hypothesis 3 (H3)*. Different learning modes are associated with different mental health statuses in university students.

*Hypothesis 4 (H4)*. Education modes may moderate the relationship between PSU and mental health problems.

## Materials and Methods

### Participants and Ethics

Participants were 1,629 full-time university students recruited from eight colleges in Beijing, Shanghai, Xi'an, Zhejiang, and Jiangxi province. The Ethics Committee of Beijing Hospital approved the study (2020BJYEC-231-01). Respondents provided electronic signatures of informed consent in the web-based survey system before the investigation. The size of the sample population was selected based on the following criteria: As recommended by some scholars, a sample item ratio >20 and sample independent variable ratio >10 are sufficient (33). Thus, the appropriate recommended sample size was 200, and we successfully collected data from over 1,000 participants.

### Procedures

A web-based online survey was conducted between March 2020 and October 2021. Well-trained student counselors from each university were responsible for sending and receiving the questionnaires, and all respondents volunteered to participate in the study. In the online survey system, subjects were not allowed to submit the online questionnaire until they had completed all the questions. A total of 1,780 questionnaires were collected; 55 questionnaires with a response time <5 min and 21 questionnaires with unidentifiable demographic information were excluded. Moreover, if all questions in the four scales were answered in an identical or repetitive manner, the questionnaire was identified as invalid (*n* = 75). Overall, 151 invalid questionnaires were excluded, and 1,629 (recovery rate = 91.52%) valid questionnaires were included in the final analysis. We investigated students' recent learning conditions through a binary question that asked subjects to report which learning method had been used most frequently in the past 1 month. Based on their responses, subjects were divided into an online learning group and traditional face-to-face learning group. Education level was classified as bachelor, master, and doctoral. Grade was classified as graduating students or not. Residence was classified as urban and rural. Education satisfaction was assessed using a 5-point Likert scale (“bad/unsatisfied” = 1–3 points; “good/satisfied” = 4–5 points). If subjects self-reported engaging in regular exercise, they were defined as having an exercise habit, and those who self-reported occasional exercise or no exercise were defined as having no exercise habit.

### Measurement

#### Smartphone Addiction Scale-Short Version

The Smartphone Addiction Scale-Short Version (SAS-SV) developed by Kwon and colleagues was used to measure the severity of problematic smartphone use (34). The scale consists of 10 items that are scored on a 6-point Likert scale, and the summed score ranges from 10 to 60. An example question is, “I feel impatient or restless when I'm not holding my smartphone”. Subjects were asked to rate how much they agreed with each statement (“disagree” = 1 point; “agree” = 6 points). According to the thresholds recommended for adolescents, male participants with a summed score ≥31 and female participants with a summed score ≥33 were identified as having PSU. The SAS-SV has been shown to have good reliability and validity in the Chinese population (35). The Cronbach's α coefficient of the SAS-SV was 0.91 in this study.

#### 9-Item Patient Health Questionnaire

The 9-item Patient Health Questionnaire (PHQ-9) (36) is a valid self-administered depression screening tool, and was used to measure the level of depressive symptoms. The scale includes 9 items that are scored on a 4-point Likert scale to assess the presence of depressive symptoms during the last week (“not at all” = 0 points; “several days” = 1 point; “more than half the week” = 2 points; “almost every day” = 3 points). Higher scores indicate more severe depressive symptoms. The summed PHQ-9 score ranges from 0 to 27, and subjects with a total score ≥10 were defined as exhibiting depressive symptoms. A recent meta-analysis confirmed that a cutoff of 10 or above can maximize the combined sensitivity and specificity (37). The PHQ-9 showed good internal consistency, with a Cronbach's α coefficient of 0.89 in the current study.

#### 7-Item Generalized Anxiety Disorder Scale

The 7-item Generalized Anxiety Disorder Scale (GAD-7) is a self-report measurement that screens the presence of generalized anxiety disorders over the previous 2 weeks (38). The scale consists seven statements about worry or somatic tension, which are rated on a 4-point Likert scale (“not at all” = 0 points; “several days” = 1 point; “more than half the week” = 2 points; “almost every day” = 3 points). Higher scores indicate more severe anxiety symptoms. Given that the GAD-7 has a low sensitivity for detecting mild symptoms, previous studies have reported that the GDA-7's appropriate screening cutoff is ≥10 in both clinical samples and the general population (39), and its Cronbach's α coefficient was 0.93 in this study.

#### 8-Item Athens Insomnia Scale

Sleep quality was measured using the 8-item Athens Insomnia Scale (AIS-8) (40). The AIS-8 is a self-assessment psychometric instrument that includes 8 items scored on a 6-point Likert scale, with a total score of 0–24. Items assess sleep induction, awakenings during the night, final awakening, total sleep duration, sleep quality, well-being, functioning capacity, and daytime sleepiness. Individuals with a summed AIS-8 score ≥6 were classified as having insomnia symptoms (41). In this study, the Cronbach's α coefficient of the scale was 0.87.

### Statistical Analysis

Continuous variables were presented as the mean ± standard deviation (M ± SD), and categorical variables as numbers and percentages [*n* (%)]. To test whether different education modes are associated with levels of smartphone addiction problems and related mental health symptoms (H2 and H3), student's *t*-test and Chi-square test were used to compare the PSU and psychological status between online learning and face-to face learning subgroups. We used Harman's single factor method to test the common method bias, which is common in multiple self-assessment questionnaires (42). All the items in the observational measurements were included in an exploratory factor analysis. If the first factor to be extracted explains <40% of the total variation, there is no common method bias. To confirmed the positively associations between PSU levels and psychological problems (H1), a series of linear regression models were used to test the correlations between SAS-SV, PHQ-9, GAD-7, and AIS-8 scores. Multiple logistic regression analyses were used to analyze the association between PSU and the risk of depressive, general anxiety, and insomnia symptoms. Subjects without PSU were used as the reference group, and odds ratios (ORs) with 95% confidence intervals (CIs) were calculated after adjusting for potential confounders. To test the moderating role of learning modes on the relationship between PSU and mental health symptoms (H4), cross-product terms were added to each model. We also performed subgroup logistic regression analyses for participants, stratified by online learning and face-to face learning subgroups. Statistical significance was defined as a two-sided *p-*value of 0.05, and CIs were computed at the 95% level. Statistical analyses were performed using Statistic Package for Social Science version 22.0 for windows (IBM Corporation, Armonk, NY, USA).

## Results

### Demographic Characteristics

The mean age of participants was 23.85 ± 3.53 years. A total of 788 (48.37%) reported online learning to be their most frequently used learning mode, and 841 (51.63%) reported traditional face-to-face learning as their most frequently used learning mode. Among the 1,629 participants, 65.25% were female, 19.89% were graduating students, and 70.04% were urban residents; 76.98% were satisfied with the current education system, and 36.28% had exercise habits.

### PSU and Psychological Health

The mean SAS-SV score was 33.81 ± 9.81, with a PSU prevalence of 58.5%. The prevalence of depressive, anxiety, and insomnia symptoms were 25.97, 21.92, and 72.99%, respectively. As shown in Table 1, the online learning group had a higher prevalence of PSU (62.82% vs. 54.46%, *P* < 0.001), depressive symptoms (29.95% vs. 22.24%, *P* = 0.003), anxiety symptoms (25.13% vs. 18.91%, *P* < 0.001), and insomnia symptoms (75.89% vs. 70.27%, *P* = 0.006) than the face-to-face learning group.

**Table 1:** Problematic smartphone use and psychological symptoms of all 1,629 students.

**Measurements**	**Overall (*n =* 1629)**	**Online learning group (*n =* 788)**	**Face-to-face learning group (*n =* 841)**	***t*** **/χ^2^**	***P*** **-value**
SAS-SV score, (*m* ± SD)	33.81 ± 9.81	34.86 ± 10.20	32.69 ± 9.81	5.419	<0.001
PHQ-9 score, (*m* ± SD)	7.75 ± 4.56	8.15 ± 4.42	7.51 ± 4.93	3.945	0.002
GAD-7 score, (*m* ± SD)	6.72 ± 4.33	7.11 ± 4.04	6.24 ± 4.69	4.537	<0.001
AIS-8 score, (*m* ± SD)	8.39 ± 4.61	8.63 ± 4.54	7.91 ± 4.76	2.504	0.015
Problematic smartphone use, *n* (%)	953 (58.50)	495 (62.82)	458 (54.46)	19.241	<0.001
Depression symptom, *n* (%)	423 (25.97)	236(29.95)	187 (22.24)	9.123	0.003
Anxiety symptom, *n* (%)	357 (21.92)	198 (25.13)	159 (18.91)	19.728	<0.001
Insomnia symptoms, *n* (%)	1189 (72.99)	598 (75.89)	591 (70.27)	7.446	0.006

*SAS-SV, Smartphone Addiction Scale-Short Version; PHQ-9, 9-item Patient Health Questionnaire; GAD-7, 7-item Generalized Anxiety Disorder Scale; AIS-8, 8-item Athens Insomnia Scale*.

### Correlations Between SAS-SV, PHQ-9, GAD-7, and AIS-8 Scores

**Figure 1:**
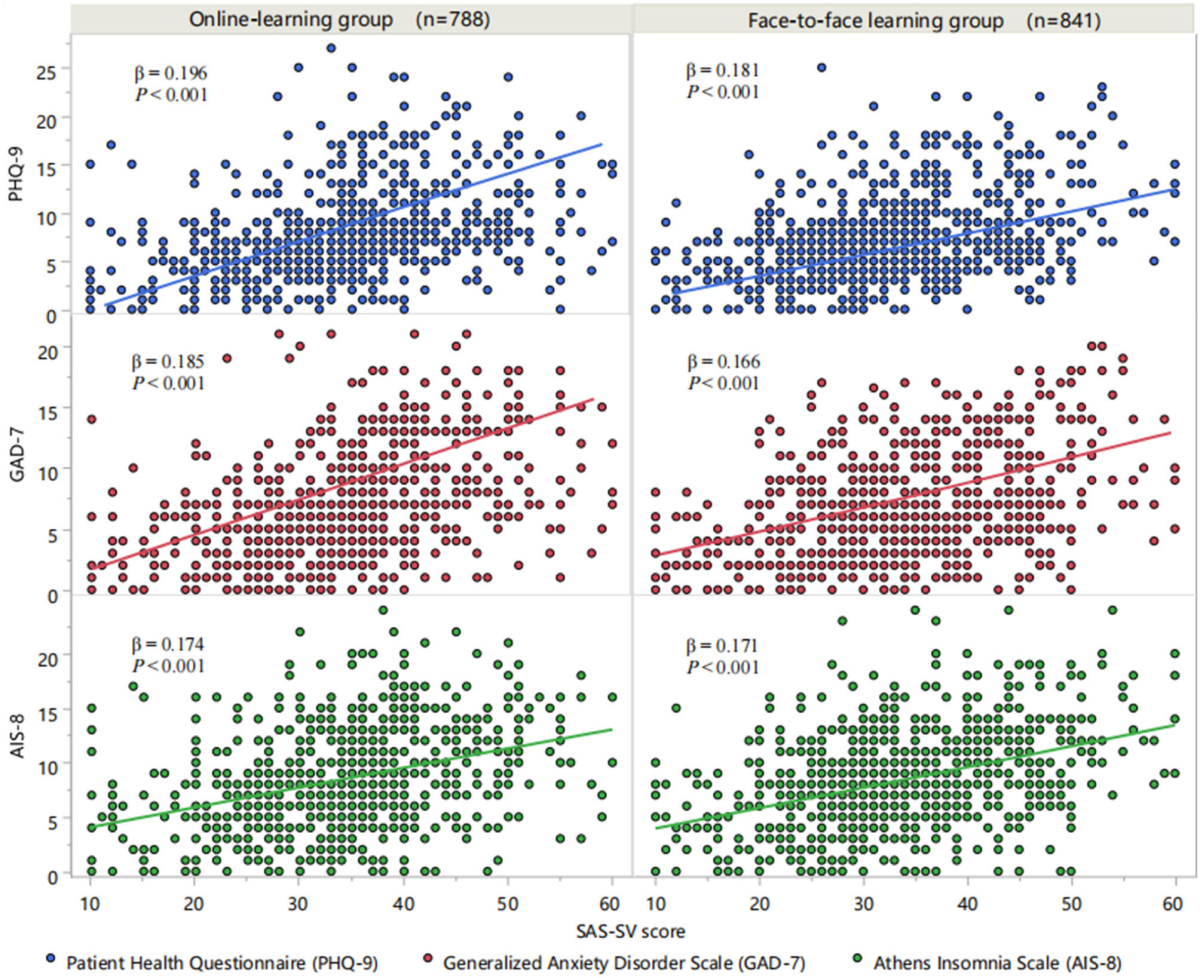
Associations between SAS-SV and PHQ-9, GAD-7, and AIS-8 scores. SAS-SV, Smartphone Addiction Scale-Short Version; PHQ-9, 9-item Patient Health Questionnaire; GAD-7, 7-item Generalized Anxiety Disorder Scale; AIS-8, 8-item Athens Insomnia Scale.

An exploratory factor analysis was performed on all 34 items in the SAS-SV, PHQ-9, GAD-7, and AIS-8, and five common factors with eigenvalues >1 were obtained. The first common factor explained 14.62% of the total variance which indicated that there was no common method bias in the current dataset. The association of the SAS-SV score with psychological measurements in the online learning group and face-to-face learning group was shown in Figure 1, respectively. As summarized in Table 2, the SAS-SV score (independent variable) and the PHQ-9, GAD-7, and AIS-8 scores (dependent variables) were included as continuous variables; education mode was a dichotomous variable. In Model 1, the SAS-SV score was significantly correlated with PHQ-9, GAD-7, and AIS-8 scores (*Ps* < 0.001). After adjusting for age, sex, grade, education level, residence, satisfaction, and exercise habits, statistical significance was also found in Model 3. To test the significance of multiplicative interaction of the SAV-SV score and education mode, we then added an interaction term (SAS-SV × education mode) to Model 2; the interaction term was statistically significant in the correlation of the SAS-SV score with the PHQ-9 score (*P* = 0.002) and GAD-7 score (*P* = 0.002), but not with the AIS-8 score (*P* = 0.426). The results were not substantially altered when covariates were adjusted in Model 4.

**Table 2:** Linear regression analysis of SAS-SV, PHQ-9, GAD-7, and AIS-8 scores.

**Dependent variable**	**Independent variable**	**Crude model 1**			**Crude model 2**			**Adjusted model 3**			**Adjusted model 4**		
		**β**	**SE**	***P*-value**	**β**	**SE**	***P*-value**	**β**	**SE**	***P*-value**	**β**	**SE**	***P*-value**
PHQ-9	SAS-SV	0.187	0.013	<0.001	0.196	0.016	<0.001	0.158	0.013	<0.001	0.195	0.017	<0.001
	online learning group	–	–	–	0.175	0.065	0.029	–	–	–	0.168	0.071	0.032
	SAS-SV × education mode	–	–	–	−0.037	0.016	0.002	–	–	–	−0.039	0.016	0.003
GAD-7	SAS-SV	0.179	0.012	<0.001	0.206	0.015	<0.001	0.162	0.012	<0.001	0.203	0.015	<0.001
	online learning group	–	–	–	0.244	0.015	0.016	–	–	–	0.291	0.155	0.026
	SAS-SV × education mode	–	–	–	−0.045	0.015	0.002	–	–	–	−0.045	0.015	0.002
AIS-8	SAS-SV	0.172	0.014	<0.001	0.181	0.017	<0.001	0.163	0.014	<0.001	0.184	0.017	<0.001
	online learning group	–	–	–	0.136	0.071	0.057	–	–	–	0.152	0.075	0.038
	SAS-SV × education mode	–	–	–	−0.013	0.017	0.426	–	–	–	−0.016	0.017	0.344

*SAS-SV, Smartphone Addiction Scale-Short Version; PHQ-9, 9-item Patient Health Questionnaire; GAD-7, 7-item Generalized Anxiety Disorder Scale; AIS-8, 8-item Athens Insomnia Scale; SE, standard error*.

*Crude Model 1: SAS-SV*.

*Crude Model 2: Model 1+ online learning group + SAS-SV × education mode*.

*Adjusted Model 3: Model 1+ age, sex, grade, education level, residence, satisfaction, and exercise habits*.

*Adjusted Model 4: Model 3+ online learning group + SAS-SV × education mode*.

### Stratified Analyses of Associations Between PSU and Psychological Symptoms

In the stratified logistic regression analyses, PSU (independent variable) and mental health symptoms (dependent variables) were included as dichotomous variables, and participants without PSU comprised the reference groups. As shown in Table 3, PSU was an independent influencing factor for depressive symptoms (AdjOR = 3.14, 95% CI = 2.26–4.37), anxiety symptoms (AdjOR = 3.73, 95% CI = 2.13–4.59), and insomnia symptoms (AdjOR = 2.96, 95% CI = 2.23–3.92), in both the crude and adjusted models. The multivariable adjusted OR of PSU for depressive symptoms in the online learning group was more pronounced than in the face-to-face learning group (OR = 4.66 vs. 2.33, *P* for interaction = 0.015). Similar results were found in the relationship between PSU and anxiety symptoms (OR = 6.05 vs. 2.94, *P* for interaction = 0.021). For the association between PSU and insomnia symptoms, no significant interaction effect was found (OR = 3.29 vs. 2.86, *P* for interaction = 0.589).

**Table 3:** Stratified logistic regression analyses of PSU and mental health symptoms across education modes.

**Dependent variable**	**Subgroup**	**No. of participants**	**No. of symptoms**	**Prevalence**	**Crude ORs^**a**^**	**Adjusted ORs^**b**^**	***P* for**
				**(%)**	**(95% CI)**	**(95% CI)**	**interaction**
Depressive symptoms	Total sample	1629	423	25.97			
	PSU	953	334	35.05	3.37(2.45-4.64)	3.14(2.26–4.37)	0.015
	Non-PSU	676	89	13.17	1	1	
	Online group	788	236	29.95			
	PSU	495	205	41.41	4.31(2.64-5.67)	4.66(2.83–6.75)	
	Non-PSU	293	31	10.58	1	1	
	Face-to-face group	841	187	22.24			
	PSU	458	129	28.17	2.39(2.38-4.85)	2.33(2.31–4.82)	
	Non-PSU	383	58	15.14	1	1	
Anxiety symptoms	Total sample	1629	357	21.92			
	PSU	953	264	27.70	3.51(2.42-5.07)	3.73(2.13–4.59)	0.021
	Non-PSU	676	93	13.76	1		
	Online group	788	198	25.13			
	PSU	495	163	32.93	5.67(3.46-9.09)	6.05(3.49–9.67)	
	Non-PSU	293	35	11.95	1	1	
	Face-to-face group	841	159	18.91			
	PSU	458	101	22.05	3.09(2.04-4.67)	2.94(1.93–4.49)	
	Non-PSU	383	58	15.14	1	1	
Insomnia symptoms	Total sample	1629	1189	72.99			
	PSU	953	761	79.85	2.97(2.27-3.88)	2.96(2.23–3.92)	0.589
	Non-PSU	676	428	63.31	1	1	
	Online group	788	598	75.89			
	PSU	495	415	83.84	3.24(2.87-5.35)	3.29(2.87–5.72)	
	Non-PSU	293	183	62.46	1	1	
	Face-to-face group	841	591	70.27			
	PSU	458	346	75.55	2.87(2.13-3.87)	2.86(2.11–3.89)	
	Non-PSU	383	245	63.97	1	1	

*PSU, problematic smartphone use; OR, odds ratio*.

a *Non-adjusted model*.

b *Adjusted for age, sex, grade, educational level, residence, satisfaction, and exercise habits*.

*Participants with no PSU comprised the reference groups*.

## Discussion

This study found significant correlations between online learning modes, smartphone addiction problems, and multiple mental health symptoms after the COVID-19 outbreak in a representative student sample in China. The high prevalence of PSU (58.5%) was comparable to that of previous studies conducted during the COVID-19 pandemic. According to a recent global survey, about 70% of Internet users around the world, especially the younger generation, were using smartphones or mobile phones more frequently as a result of the COVID-19 outbreak (13). Another cross-sectional study reported a PSU prevalence of 43.3% among an adult sample from Macao, China (43). In the current study, the prevalence of depressive, anxiety, and insomnia symptoms were 25.97, 21.92, and 72.99%, respectively. Similarly, in a recent study conducted in East China, 35.15, 36.32, and 17.24% of college students studying completely online at home during the COVID-19 pandemic reported symptoms of depression, anxiety, and stress, respectively (44). A recently published paper reported that after the COVID-19 pandemic, the estimated number of people with major depression increased from 193 million to 246 million globally, and the number of people with anxiety increased from 298 million to 374 million (45). A recent meta-analysis reported that the pooled prevalence of anxiety symptoms and of depressive symptoms in Chinese college students were 31 and 34%, respectively (46). These findings indicate that mental health disorders, especially sleep problems, remained serious among university students during the “new normal” of the COVID-19 pandemic.

The prevalence of PSU (62.82% vs. 54.46%) was higher in the online learning group than in the traditional face-to-face learning group, as were symptoms of depression (29.95% vs. 22.24%), anxiety (25.13% vs. 18.91%), and insomnia (75.89% vs. 70.27%). The significant differences between the online and face-to-face learning groups could be explained in several ways. First, online learning is a model that uses electronic technology and media to provide instructional teaching support (11). As a direct result, students increase the frequency of smartphone use and access, which in turn increases the potential risk of overuse. Many hours of online study can also lead to physical symptoms, which are closely linked to psychological problems (47). Second, college students who study in a more isolated online environment experience more study pressure and confusion than students who study intensively in the classroom. Compared to centralized learning, this model of learning lacks opportunities for interaction, such as face-to-face cooperative learning and on-site discussion; this may reduce social and peer support, and thus augment symptoms of depression and anxiety. In another study, college students learning remotely were found to be more isolated and to have a lower level of self-confidence (48). Third, during the transition to online learning, student satisfaction may decrease due to problems such as disorganized teaching activities and imperfect teaching platforms. Satisfaction with the teaching environment and quality may also be associated with university students' mental health problems. Similar to the present study, Bolatov found an increase trend of depression, anxiety, and burnout symptoms in Kazakhstan medical students after the transition from traditional learning to online learning (49). Another Polish study also showed that a completely online learning mode had a negative impact on the mental health of college students during the COVID-19 pandemic (50). In turn, a longitudinal study from the US found the opposite with a gradual alleviation in COVID-19-related stress and sleep problems of college students when transitioning to online learning mode (41). These inconsistent results may be explained by the radically different epidemic situations and lockdown measures between the US and China, which could have resulted in different attitudes toward and participation in online learning in college students.

The multiple regression analysis showed that subjects with PSU were more likely to report depressive symptoms (AdjOR = 3.14, 95% CI = 2.26–4.37), anxiety symptoms (AdjOR = 3.73, 95% CI = 2.13–4.59), and insomnia symptoms (AdjOR = 2.96, 95% CI = 2.23–3.92). These findings are consistent with previous studies in adolescents (51, 52) and college students (53, 54). A cross-sectional study with adult US participants found a positive relationship between the time spent online searching for information about COVID-19 and GAD-7 scores (55). In Hao et al.'s study, academic burnout, anxiety, and resilience were significantly associated with PSU in Chinese undergraduate students during the pandemic (56). Furthermore, we found that the association of PSU with depressive symptoms (OR = 4.66 vs. 2.33, *P* for interaction = 0.015) and with anxiety symptoms (OR = 6.05 vs. 2.94, *P* for interaction = 0.021) was more pronounced in the online learning group. This unique interaction further strengthened the potential negative effect of the online education mode on mental health status during the COVID-19 epidemic. This finding suggested that college students who participate in online learning might be more prone to depression or anxiety symptoms when they developed smartphone addiction problems. The moderating role of education mode on PSU and psychological problems has not been previously reported; this result is therefore of great value for the future development of targeted interventions and psychological counseling for specific student groups. The I-PACE model and CIUT could support the associations between PSU with depression and anxiety. Namely, the outbreak of COVID-19 and the sudden change in learning patterns can be regarded as an abnormal change in the environment; thus, students who are in a negative or worried mood are more inclined to seek mental compensation from mobile phones or the Internet. In addition, excessive use of smartphones is conducive to addiction to the virtual world, which can result in social phobia and loneliness, and can trigger or aggravate depression (57). We argue that the effects of smartphones on mental health need to be vigilantly monitored in students learning online. Timely educational monitoring and targeted psychological interventions are necessary to maintain the new normal of distance learning in the post-COVID-19 era. For example, occupational psychological therapies have shown potential effects in alleviating psychological distress and sleep problems (58). Besides, some clinical mechanisms are beneficial to support the associations of psychological disorders and PSU. Inhibition of melatonin secretion caused by nocturnal light stimulation is a recognized physiological pathway that leads to sleep deprivation. Radio frequency electromagnetic fields generated during the use of mobile phones can affect the normal blood flow of the brain and metabolic function of the body, thus exacerbating emotional disorders and inhibiting slow-wave sleep (59).

## Limitations

Several limitations of the current study should be acknowledged. First, no causal inferences can be made due to the study's cross-sectional design. Longitudinal research should be conducted in the future. Second, although the web-based survey was the best option to reach subjects during the COVID-19 epidemic period, the response bias was inevitable. Third, smartphone addiction and mental health-related information were collected from self-report questionnaires, which were less reliable than clinical diagnoses. Our future direction is the application of objective measurements, as some previous studies have done (25, 60).

## Conclusions

Our study provides evidence that Chinese university students have serious smartphone addiction problems that are associated with depressive, anxiety, and insomnia symptoms. Online learning was found to exacerbate PSU and mental health problems. This suggests that attention should be paid not only to the organization of teaching activities, but also to students' mental health status.

## Data Availability Statement

The original contributions presented in the study are included in the article/Supplementary Material, further inquiries can be directed to the corresponding author/s.

## Ethics Statement

The studies involving human participants were reviewed and approved by the Ethics Committee of Beijing Hospital. The patients/participants provided their written informed consent to participate in this study.

## Author Contributions

CZ and HY proposed the concept and design. CZ and JC analyzed and interpreted the data and wrote the manuscript. JH, YL, and HY drafted and edited the manuscript. CZ, JC, and HY supervised the study and obtained funding. All authors read and approved the final version of the manuscript.

## Funding

This study was supported by the National Key R&D Program of China (2020YFC2002700); the Education and Teaching Research Project of Peking University Health Science Center (2020YB42), and the Fundamental Research Funds for the Central University (3332021077). We sincerely thank all investigators and students who participated in this study, for their joint effort and cooperation.

## Conflict of Interest

The authors declare that the research was conducted in the absence of any commercial or financial relationships that could be construed as a potential conflict of interest.

## Publisher's Note

All claims expressed in this article are solely those of the authors and do not necessarily represent those of their affiliated organizations, or those of the publisher, the editors and the reviewers. Any product that may be evaluated in this article, or claim that may be made by its manufacturer, is not guaranteed or endorsed by the publisher.
